# *FMR1* gene CGG repeat distribution among the three individual cohorts with intellectual disability, autism, and primary ovarian insufficiency from Tamil Nadu, Southern India

**DOI:** 10.1002/ggn2.10048

**Published:** 2021-05-28

**Authors:** Indhumathi Nagarathinam, Samuel S. Chong, Thelma B. K., Jeffrey Justin Margret, Viswanathan Venkataraman, Karthikeyen Natarajan Padmavathy, C. R. Srikumari Srisailapathy

**Affiliations:** ^1^ Department of Genetics, Dr. ALM Post Graduate Institute of Basic Medical Sciences University of Madras Chennai India; ^2^ Department of Medical Genetics, Laboratory Services, Apollo Main Hospital Chennai India; ^3^ Department of Pediatrics, Yong Loo Lin School of Medicine National University of Singapore Singapore Singapore; ^4^ Department of Obstetrics and Gynaecology, Yong Loo Lin School of Medicine National University of Singapore Singapore Singapore; ^5^ Department of Genetics University of Delhi New Delhi India; ^6^ Kanchi Kamakoti Childs Trust Hospital Chennai India; ^7^ Apollo Children's Hospital Chennai India; ^8^ DOAST (Doctrine Oriented Art of Symbiotic Treatment) Hearing Care Center and Integrated Therapy Center for Autism Chennai India

**Keywords:** autism spectrum disorder, CGG repeat variation, fragile X syndrome, intellectual disability, primary ovarian insufficiency

## Abstract

Fragile X syndrome is the most common genetic cause of intellectual disability (ID) and is also well known to have a role in primary ovarian insufficiency (POI) and fragile X‐associated tremor ataxia syndrome (FXTAS) that expresses across generations. The objective was to compare the CGG repeat variants in *FMR1* gene among three correlating cohorts of ID, autism and idiopathic POI. Thirty‐six patients with ID, 12 with autism spectrum disorder (ASD) and 13 females with idiopathic POI were screened for *FMR1* CGG repeat size by fluorescent methylation‐specific PCR and GeneScan analysis, irrespective of Hagerman checklist clinical scores. Among 29 males and seven females, 11 *FMR1* allelic variants ranging from 21 to >200 CGG repeats were observed. Three (CF2‐3, 39‐5, 44‐2) out of 29 males had full mutation alleles accounting for a 10.34% incidence of FXS among idiopathic ID males. One of them was a mosaic for CGG repeats with both premutation and full mutation alleles. The frequency of fragile X syndrome is high among patients with idiopathic ID; they also had a high score for the clinical check list. A cascade testing that begins with checklist evaluation prior to DNA analysis will be cost‐effective for establishing early diagnosis in South India. With the huge disease burden, there is a need for the establishment of more molecular diagnostics and self‐help groups for fragile X syndrome.

## Graphical abstract

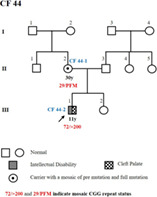

## 1 INTRODUCTION

Fragile X syndrome (FXS, MIM 300624), is the most common genetic cause of intellectual disability (ID) and one of the leading genetic causes of autism with a prevalence of one in 7000 males and one in 11 000 females.^1^ It is caused by an expansion of a CGG repeat above 200 units in exon 1 of 5′ UTR and subsequent methylation induced silencing of the fragile X mental retardation‐1 (*FMR1*) gene at Xq27.3.

The CGG triplet is usually composed of 5‐44 repeats (normal) or 45‐54 repeats (intermediate or gray zone—GZ), allowing transcription and translation of the *FMR1* gene. Normal alleles are usually transmitted stably over generations. Intermediate alleles may or may not show intergenerational instability and are considered a possible risk factor for the CGG repeat expansion.^2^ When the CGG repeat expands between 55 and 200 (premutation—PM), the gene continues to transcribe (more) mRNA even though the premutated repeats become meiotically unstable.^3^ If the repeats exceed 200 (full mutation—FM) it results in hypermethylation of the CGG repeat and adjacent promoter region, transcriptional silencing, and the consequent loss of FMR1 protein (FMRP).^4^ FMRP is an RNA binding protein that controls the transport and translation of its target mRNAs in brain and is important for synaptic plasticity.^5^ Involvement in alternative splicing, RNA editing and DNA repair are the other roles implicated for FMRP.^6^ Concerted efforts in understanding FMRP functions have identified critical pathways altered in the absence of FMRP.^7^

FXS is characterized by a broad spectrum of behavioral, cognitive and physical impairments due to lack of FMRP.^4^ Behavioral manifestations in FXS are quite variable and include attention deficits, hyperactivity/impulsivity, hyper‐arousal, anxiety, self‐injurious behavior, and autism spectrum disorder (ASD). ASD is a developmental disorder with multiple etiologies, and is characterized by impairment in social interaction and communication, and the presence of restricted and repetitive patterns of behavior, interests, or activities. The degree of cognitive impairment ranges from normal intelligence with learning disabilities to intellectual disabilities.^8^

FXS is also associated with risk for deficits in other domains, including executive function, sustained attention, working memory, and social function even in those without mental retardation.^4^ Prevalence studies in India, of FXS among ID individuals are summarized in Table 1, ranging between 2.5 and 12.24%.

**TABLE 1:** Studies reporting prevalence of fragile X syndrome in those with ID in the Indian population

S. no	Ethnicity	Sample size	Methodology	Frequency	References
1.	South India	98	PCR; Southern blotting	7.14%	^29^
2.	Delhi	360 (M)	MS PCR; Southern blotting	5.27%	^30^
3.	Calcutta	98	PCR; Southern Blotting	7%	^31^
4.	New Delhi	130 (93 M & 37 F)	Radioactive PCR; Southern Blotting	7.7%	^32^
5.	Uttar Pradesh	146 (118 M & 28 F)	PCR; Southern blotting	2.54%	^33^
6.	Karnataka	25 (M)	MS PCR; Southern blotting	4%	^34^
7.	North India	294 (271 M & 10 F)	PCR; Southern blotting	12.24%	^35^
8.	Calcutta	157 NBD (112 M & 45 F)	MS PCR; Southern Blotting	3.178%	^36^
9.	Andhra Pradesh	337 (316 M & 21 F)	Radioactive PCR	3.6%	^37^
10.	Uttar Pradesh	59	TP‐PCR; MS‐PCR	10%	^9^
11.	Lucknow, Chandigarh, Jodhpur and Pondicherry	233 (218 M & 15 F)	TP‐PCR	7.7%	^38^
12.	Tamil Nadu	36 (29 M & 7 F)	MS‐PCR	10.34%	Present study

Abbreviations: ID, intellectual disability; NBD, neurobehavioral disorders; TP‐PCR, triple primed PCR.

Individuals with a PM allele will be asymptomatic but may develop late onset conditions such as fragile X‐associated tremor ataxia syndrome (FXTAS, MIM 300623) in both males and females, or fragile X‐associated primary ovarian insufficiency (FXPOI, MIM 311360) in females. Moreover, PM females are at a high risk of having a FXS child.^9^ The frequency of PM carriers among POI women is 12.9%.^10^ The risk of developing POI in PM positive females is 20% to 28% depending on the size of the CGG repeat length, with the greatest risk for 80 to 100 CGG repeats. Thus, women with a family history of POI or elevated levels of follicle‐stimulating hormone (FSH) before the age of 40 years without a known cause should be tested to rule out PM positivity as a cause of ovarian insufficiency.^11^

Thus, this study compares the CGG repeat variation in the *FMR1* gene among three correlated cohorts: (1) intellectual disability, (2) autism, and (3) women with primary ovarian insufficiency using a methylation‐specific PCR (ms‐PCR) assay.

## 2 RESULTS

A total of 43 X chromosomes from 36 individuals with ID were analysed. Repeat sizes 29 and 30 were the most frequent, accounting for 66% and 75% of chromosomes in males and females, respectively (Table 2). No intermediate or premutation repeats were observed in this cohort. None of the seven affected females had full mutations. Three of the 29 affected males (10.34%) had full mutations (≥200 repeats) and their families are detailed below.

**TABLE 2:** Frequency distribution of chromosomes by CGG repeats among idiopathic ID

CGG repeat number	Males		Females	
	*N*	%	*N*	%
21	1	3.45	0	0
23	1	3.45	0	0
24	2	6.90	0	0
25	1	3.45	0	0
29	10	34.48	6	42.86
30	9	31.03	5	35.71
31	1	3.45	1	7.14
32	0	0	1	7.14
34	1	3.45	0	0
38	0	0	1	7.14
≥200	3	10.34	0	0
Total	29	100	14	100

**FIGURE 1:**
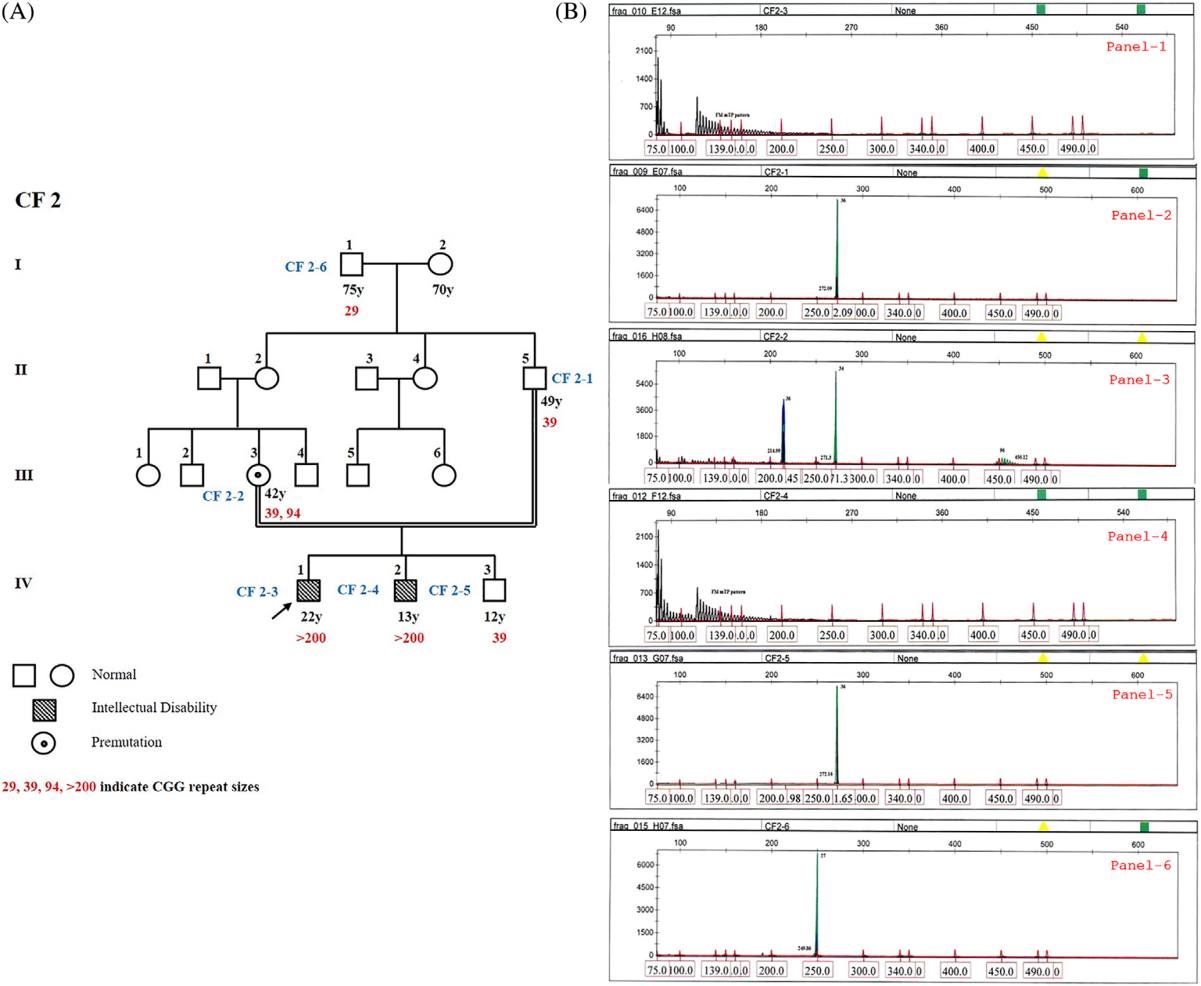
A, Four generation pedigree of proband, CF2‐3. B, GeneScan results from fluorescent ms‐PCR assay for CF2 family. The proband CF2‐3 (Panel 1) and his affected brother CF2‐4 (Panel 4) were found to have FM allele (>200); proband's mother CF2‐2 (Panel 3) has a normal and PM allele (20/94); proband's father CF2‐1 (Panel 2), normal sib CF2‐5 (Panel 5) and paternal grandfather CF2‐6 (Panel 6) were found to have normal alleles. GeneScan results generated by mTP‐PCR (black patterns), Met‐PCR (blue peaks), and nonMet‐PCR (green peaks) and Rox‐labeled internal size calibrator (red peaks)

The first case is a 22‐year‐old full mutation male proband (CF2‐3; IV‐1) born to uncle‐niece parents (Figure 1A). Based on the Wechsler Adult Performance Intelligence Scale his IQ was 45 (moderate ID) and the Hagerman Fragile X Checklist (HFXC) score was 19 (Table S2A). Speech and developmental milestones were delayed, with diagnosis of ID at 4 years. Elongated face, broad forehead, large ears, prominent jaw, hyper‐extensible fingers and macro‐orchidism were observed. Stereotypic behaviors, shyness, self‐talking, short attention span, poor eye contact, hand‐flapping, head banging, and self‐mutilation were also reported. Sleep pattern was normal with no history of seizures. His affected brother (CF2‐4; IV‐2) (>200 repeats) (Figure 1B; Panel‐4), had similar clinical features in addition to thick lips and dental crowding, with an IQ of 40 and HFXC score of 21 (Table S2C). His normal sib (CF2‐5; IV‐3) has a 39 repeat allele (Figure 1B; Panel‐5). His asymptomatic mother's (CF2‐2; III‐3) genotype is 39/94 (normal/premutation alleles) (Figure 1B; Panel‐3). His paternal grandfather (CF2‐6; I‐1) and father (CF2‐1; II‐5) had normal CGG repeats of 29 and 39, respectively, while his maternal grandfather was unavailable for genotyping.

**FIGURE 2:**
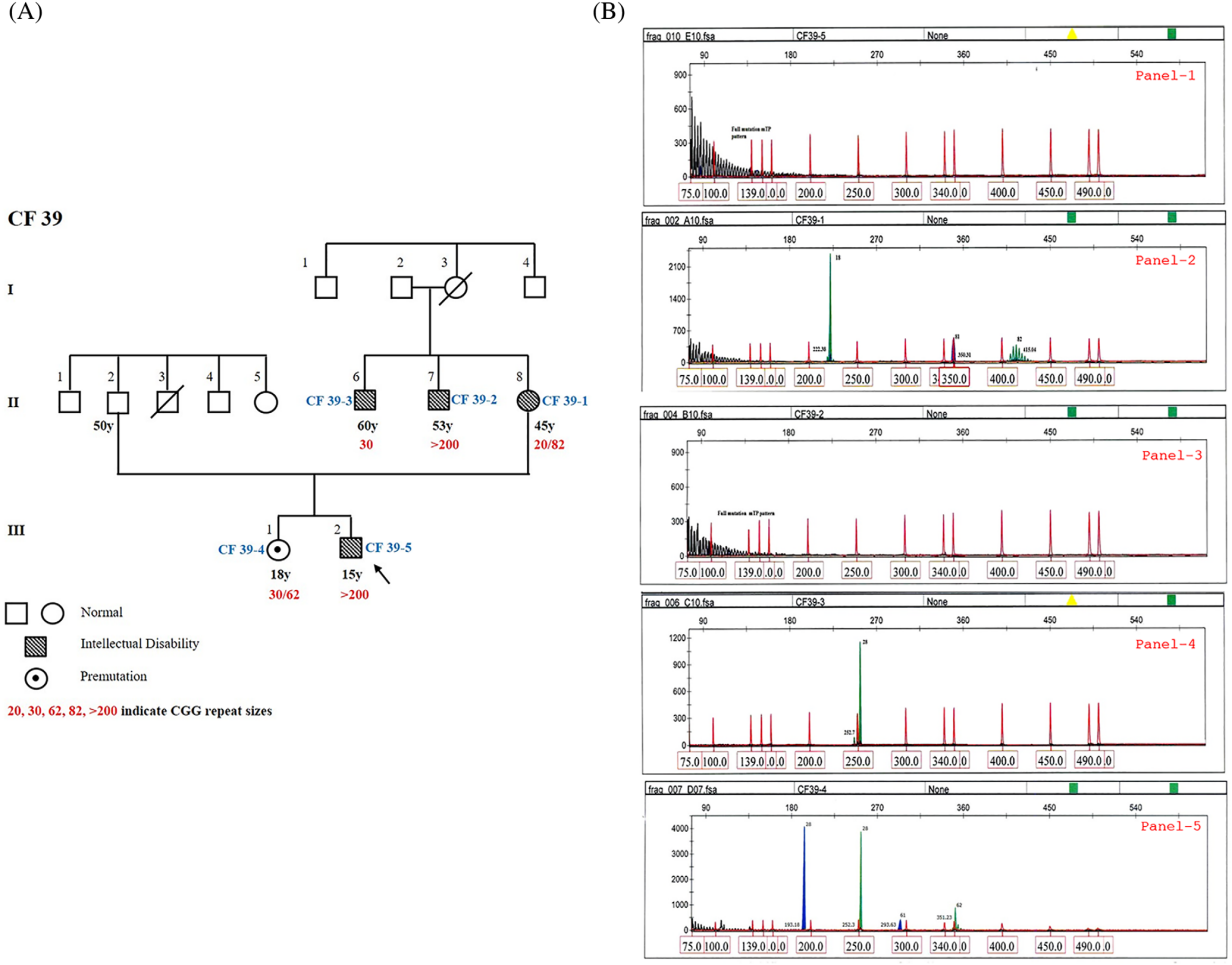
A, Three generation pedigree of proband, CF39‐5. B, GeneScan results from fluorescent ms‐PCR assay for CF39 family. The proband CF39‐5 (Panel 1) and his affected younger maternal uncle CF39‐2 (Panel 3) were found to have FM allele (>200); proband's affected mother CF39‐1 (Panel 2) his asymptomatic elder sister CF39‐4 (Panel 5) have both normal and PM allele; proband's affected elder maternal uncle CF39‐3 (Panel 4) has a normal allele

The second case is a 15‐year‐old male proband (CF39‐5; III‐2) born to non‐consanguineous parents, with a family history of FXS (Figure 2A) and carries a full mutation (Figure 2B; Panel‐1). He has moderate ID (IQ = 47) and a HFXC score of 19 (Table S2A). He had delayed milestones, diagnosed at the age of 1.5 years. His social maturity was that of an 11‐year‐old (Vineland Social Maturity Scale). Broad forehead, large ears, strabismus, prognathism, flat feet, increased muscle tone, hyper‐extensible fingers and macroorchidism were observed. Shyness, short attention span, poor eye contact, hand‐flapping, and hypersensitivity to sounds, new places and situations were observed. Sleep was impaired with no history of seizures. He showed specific interest in music. His asymptomatic sister (CF39‐4; III‐1 in Figure 2A) is a PM carrier with 30/62 repeats (Figure 2B; Panel‐5). His mother (CF39‐1; II‐8) has a PM carrier genotype (20/~82 repeats) (Figure 2B; Panel‐2) but is affected, with a HFXC score of 13 (Table S2C). Her social maturity is that of a 15‐year‐old (VSM Scale). She has an elongated face, prominent low‐set ears, dental crowding, irregularities in the lower jaw, increased crease markings in all four limbs, stereotypic behavior and emotional instability.

The proband's two maternal uncles (CF39‐2, CF39‐3) have ID with similar facial characteristics and macroorchidism. However, one of them (CF39‐2; II‐7, 53 years) carries a FM allele of >200 repeats with severe ID (Figure 2B; Panel‐3), while the other uncle (CF39‐3; II‐6, 60 years) has only a normal allele of 30 CGG repeats (Figure 2B; Panel‐4); his intellectual disability despite not having a FM allele merits further investigation for other psychiatric disorders to achieve finer resolution of the phenotype. CF39‐1 and CF39‐3 might be sharing a non CGG expansion genetic cause for their shared phenotypes. Similarly, the proband's PM carrier sibling CF39‐5 should be followed up in future for any FXPOI and FXTAS manifestations with age.

**FIGURE 3:**
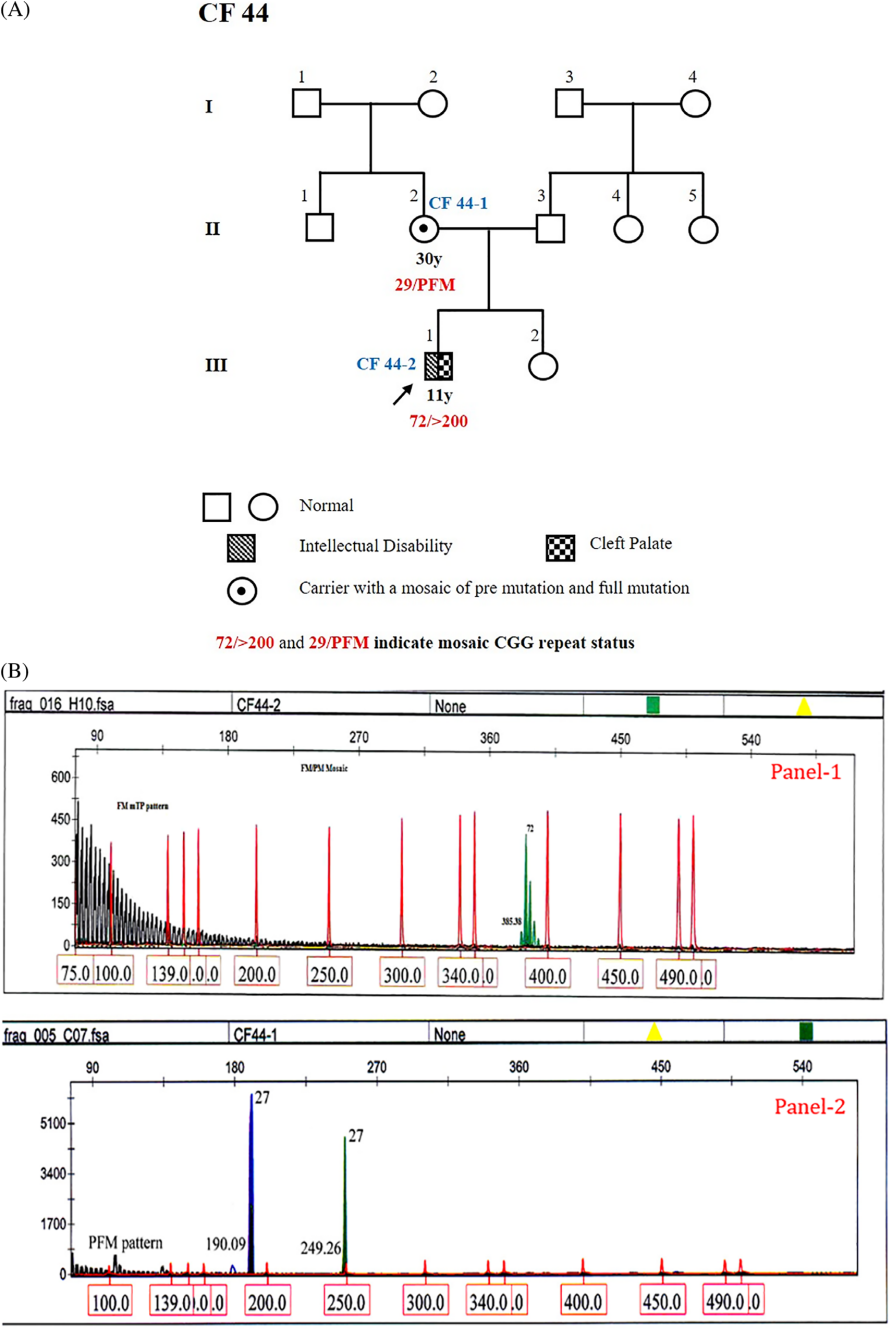
A, Three generation pedigree of proband, CF44‐2. B, GeneScan results from fluorescent ms‐PCR assay for CF44 family. The proband CF44‐2 (Panel 1) was found to have mosaic CGG repeat PM and FM alleles (72/>200); proband's mother CF44‐1 (Panel 2) have normal and a mosaic of premutation and full mutations alleles (29/164 or >200)

The third case is a sporadic 11‐year‐old male proband (CF 44‐2; III‐1), born to non‐consanguineous parents (Figure 3A). He has mild ID (IQ = 65) and a HFXC score of 22 (Table S2A) with congenital incomplete clefting of the secondary palate and normal developmental milestones but with speech dysfluency and misarticulation of syllables. He was diagnosed with ID at the age of 4 years, and showed mosaic status for PM and FM alleles (72/>200) (Figure 3B; Panel‐1). The social maturity is that of an eight‐year‐old (VSM Scale). Physical characteristics include elongated face, broad forehead, large ears, prominent jaw, flat feet, increased muscle tone, hyperextensible fingers and increased crease markings. Misarticulation of speech, echolalia, shyness, short attention span, poor eye contact, hand‐flapping, hyperactivity, were observed. He had highly impaired sleep pattern with hypersensitivity to sound, and a history of seizures. But sedated sleep EEG showed no epileptic activity. His mother (CF 44‐1; II‐2) has both a normal allele and a mosaic of premutation and full mutation alleles that is notated as PFM (pre/full mutation) genotype (29/164 or > 200) (Figure 3B; Panel‐2). However, she did not have any clinical manifestation.

Among the 58 unaffected relatives screened, males (n = 21) had CGG repeats ranging between 23 and 44. However, among the unaffected females screened (n = 37), about 4.05% (3/74) of the chromosomes have a premutation and 1.35% (1/74) of the chromosomes showed pre/full mutations (PFM). Intermediate alleles were absent (Table S4). The HFXC scores among FXS probands varied (19‐21) with a mean score of 20. Affected relatives have a mean score of 18.5 (Table S2C). A comparable decline in the triplet repeat sizes of the affected relatives may be a reason for this difference in the score.

Fifteen X chromosomes from 12 ASD patients were genotyped. Two affected sibs (one male and one female) and 16 unaffected relatives (five males and 11 females) were also included. Only normal repeat ranges were observed in both sexes (28‐30). Neither PM nor FM alleles were observed (Table S5).

Thirteen females with idiopathic POI and their four affected relatives (three sibs, and one mother) were genotyped. CGG repeat variants 29 and 30 account for about 73.5% of the chromosomes analyzed. No intermediate, PM or FM alleles were observed (Table S6).

## 3 DISCUSSION

Expansion of CGG repeats in the *FMR1* gene is a leading cause of ID in males and POI in females and is also associated with ASD.^11^ With an overall frequency of 8.34% among all ID individuals the range of repeat variation was much greater in males (21 to ≥200) than in females (29 to 38). The frequency of FXS ranges between 2.5% and 12.24% in Indians (Table 1) and 0.3% and 17% in world population studies with unexplained ID (Table S1), that may be attributed to different clinical phenotypes/founder effect. In a more recent screening study of 674 cases from Iran using traditional and triple repeat primed PCR methods, 1.1% and 9.7% of ID males had a PM or FM allele, respectively, while 0.66% were mosaics for PM and FM alleles. Furthermore, 1.9% and 5.8% of ID females were GZ and PM carriers, respectively.^12^ It is hypothesized that the founding group over‐represents relatively unstable alleles in the intermediate or premutation range that manifests over the passage of time in the progenies.^13^

Typically, the frequency observed in the Chennai population exceeds other reported studies from different states of South India which may be attributed to the increased coverage and precise sizing achieved by the ms‐PCR and GeneScan analysis approach used. Although Southern blot analysis detects large expansion mutations reliably, small premutation alleles cannot be distinguished from normal alleles in the high end of the range.^14^

Clinical features showed both intra and inter family variance. The HFXC mean scores in ID are higher in FXS positive (20) compared to negative cases (10.76). Thus, the HFXC scores enhance our ability to detect FXS among individuals with ID. Among the three affected males, one (33.33%) exhibited size mosaicism for CGG repeats (Figure 2B; Panel‐1) with PM and FM alleles (72/>200). Mosaicism associating full mutation and premutation alleles is not uncommon and has been observed in more than 40% of affected males.^15^ Nolin et al.^16^ studied 61 FXS mosaic males and found 82% with mosaic full mutation/premutation. In the early embryonic stage before methylation, fully expanded repeats experience mitotic instability, frequently leading to size mosaicism within or across cell types.^17^ The spectrum of mosaicism can be further complicated by the presence of deletions in the *FMR1* gene ranging from 1 bp to several Mbp.^18^

Among 12 ASD probands and their two affected relatives, no CGG expansions were observed. Similarly, Otsuka et al.^19^ analyzed 109 ASD Japanese patients but found no expanded alleles. However, Madrigal et al.^20^ found 1.3% of intermediate repeat expansion in ASD patients. A number of genetic disorders can lead to autism without FXS; therefore, the search for additional abnormalities in chromosomes associated with ASD may be pertinent. Although co‐morbidity rates vary by study, there is no disputing that there is an overlap in some behaviors exhibited by individuals with FXS and those with ASD.^21^

Neither the 13 POI probands nor their four affected relatives showed any CGG expansion in our study. Since the sample size is small, we are not able to come up with a confident statistical correlation. A review of several screening studies of POI cohorts shows that FX premutation occurrence in familial cases to be high (9.5%) compared to sporadic cases, where the reported frequency is 3%.^22^, ^23^ Approximately 20% of premutation carriers develop FXPOI.^24^ Timely information about the individual's carrier status will help them make informed reproductive decisions in the light of the genetic risk.^11^ However, in our earlier study from the same region, screening of 705 normal women for premutation showed a carrier frequency of one in 353 (0.14%).^25^

We observed a high incidence of FXS FM expansions (10.34%) among ID affected males in our study, highlighting the need to carry out fragile X testing among idiopathic ID individuals in this region. The HFXC scores are higher in fragile X positive cases than in negative cases. Hence higher Hagerman scores can be used as a marker to detect FXS among ID individuals. More recently Lubala et al^26^ developed a simplified universal checklist. Subjects with intellectual disability, with or without fragile X mutation were studied. The highest risk features were determined as velvet palm with redundancy of skin on the dorsum, large testes and ears, family history, autistic like behavior, flat feet and plantar crease. Thus, a cascade testing that begins with clinical checklist evaluation prior to DNA analysis will be cost‐effective for establishing early diagnosis for highly populated developing countries like India.

## 4 MATERIALS AND METHODS

### 4.1 Ethical review and informed consent

All subjects were ascertained from several hospitals in Chennai and this study was approved by the University of Madras institutional ethical review committee. Written informed consent was obtained from the unaffected family members, participants and parents or guardians on behalf of the minors or for those with ID and autism.

### 4.2 Research participants

Cohort I—36 intellectually disabled probands (29 males; seven females) and their eight affected and 58 unaffected relatives.

Cohort II—12 autism spectrum disorder (ASD) (nine males; three females) and their two affected and 16 unaffected relatives. All affected members in both cohorts were administered the Hagerman Fragile X score checklist (http://www.fragilex.org/html/checklist.htm). A score of: 0 indicates absent clinical features, 1 indicates borderline clinical features, and 2 indicates clinical features to be definitely present (Tables S2A‐C).

Cohort III—13 females with idiopathic primary ovarian insufficiency and their four affected relatives. POI status is defined as cessation of ovarian function for >6 months, before age 40 years, and with elevated follicle stimulating hormone (≥40 IU/L). Menstrual, reproductive and hormonal profiles of idiopathic POI probands are shown in Table S3.

Genomic DNA was extracted from 5 to 10 ml of whole blood by the salting‐out method.^27^ *FMR1* CGG repeat size was determined by fluorescent methylation‐specific polymerase chain reaction (ms‐PCR) and GeneScan analysis. DNA samples were treated with sodium bisulfate.^14^ Three sets of primers were used to amplify the antisense strand of bisulfite modified DNA, one set targeting the non‐methylated allele—nonMet‐PCR and the two other sets targeting the methylated allele—Met‐PCR and “mTP‐PCR.” mTP‐PCR is an adaptation of the triplet‐primed PCR (TP‐PCR) strategy of Warner et al.^28^

Amplified products of mTP‐PCR, Met PCR and nonMet PCR reactions were pooled in the ratio of 6:1:1, respectively. A 4 μl aliquot of the combined ms‐PCR products were diluted with 9 μl of HiDi Formamide and 0.5 μl of Map Marker Rox‐500 size marker (Applied Biosystems, Foster City, CA) and denatured for 5 min at 95°C. The denatured ms‐PCR aliquot was capillary electrophoresed on the ABI Prism 3100 Genetic Analyzer with an injection time of 22 s at 1KV for 40 min to size the CGG repeats. Fragment size analysis was carried out using GeneMapper software with run module Frag36_POP4_D. GeneMapper software is a flexible genotyping software package that provides DNA sizing and quality allele calls for electrophoresis‐based genotyping systems. Schematic representations of normal, premutation and full mutation ms‐PCR products by Gene Scan fragment analysis are shown in Figure S1.

## CONFLICT OF INTEREST

The authors declare no conflict of interests.

## AUTHOR CONTRIBUTIONS

C. R. Srikumari Srisailapathy designed the study; Nagarathinam Indhumathi and C. R. Srikumari Srisailapathy conducted the field work; Nagarathinam Indhumathi, Samuel S. Chong and B. K. Thelma carried out all the experiments and analyzed the data; Venkataraman Vishwanathan and Natarajan Padmavathy Karthikeyen carried out the clinical investigations; Nagarathinam Indhumathi, Justin Margret Jeffrey and C. R. Srikumari Srisailapathy wrote the manuscript. All authors read and approved the final manuscript.

### PEER REVIEW

The peer review history for this article is available at https://publons.com/publon/10.1002/ggn2.10048.

## Supporting information


**TABLE S3**: Studies reporting prevalence of fragile X syndrome in the world population.
**TABLE S4**: Hagerman Fragile X Checklist scores of 36 Idiopathic ID (Cohort I)
**TABLE S5**: Hagerman Fragile X checklist scores of 12 ASD probands (Cohort II)
**TABLE S6**: Hagerman Fragile X Checklist scores of affected relatives of Cohorts I & II

Supplementary TPR File
