# Supplementary material for: FMR1 gene CGG repeat distribution among the three individual cohorts with intellectual disability, autism, and primary ovarian insufficiency from Tamil Nadu, Southern India
Source: Adv Genet (Hoboken). 2021 May 28;2(2):e10048. doi: 10.1002/ggn2.10048 (PMC9744524; doi:10.1002/ggn2.10048)
Supplement: Supplementary file 1 — TABLE S3: Studies reporting prevalence of fragile X syndrome in the world population. TABLE S4: Hagerman Fragile X Checklist scores of 36 Idiopathic ID (Cohort I) TABLE S5: Hagerman Fragile X checklist scores of 12 ASD probands (Cohort II) TABLE S6: Hagerman Fragile X Checklist scores of affected relatives of Cohorts I & II [file GGN2-2-e10048-s001.pdf]

## Supplementary Data

**Table S1**: **Studies reporting prevalence of fragile X syndrome in the world population.**

| S. No | Ethnicity | Sample Size | Methodology | Frequency | Reference |
|---|---|---|---|---|---|
| 1. | China | 50 | PCR | 2% | [1] |
| 2. | Iran | 508 | PCR; Southern Blotting | 6.3% | [2] |
| 3. | New Zealand | 2046 | Fluorescent PCR; Southern blotting | 0.6% | [3] |
| 4. | Marshfield | 8469 | PCR | 0.3% | [4] |
| 5. | Pakistan | 333 | MS PCR; Southern Blotting | 4.8% | [5] |
| 6. | Saudi Arabia | 63 ID (53 M & 10 F) | MS PCR; Southern blotting | 17% | [6] |
| 7. | China | 553 | TP-PCR | 0.93% | [7] |
| 8. | Pakistan | 395 | Conventional PCR; Southern Blotting | 3.3% | [8] |
| 9. | Malaysia | 1245 | MS PCR | 3.5% | [9] |
| 10. | Sri Lanka | 850 | TP PCR; MS PCR; Southern Blot | 1.3% | [10] |
| 11. | West Iran | 200 | Cytogenetic and DNA sequencing | 8% | [11] |
| 12. | Iran | 656 (449 M & 207 F) | PCR; TP PCR; MS MLPA | 10.36% | [12] |

M – Males; F – Females

**References:**


1. Wang X, Hou M, Zhang D, Zhong N. Molecular screening of FMR1 mutation among autism patients in China. *World J Pediatr* 2006; 2:285-7
2. Pouya AR, Abedini SS, Mansoorian N, Behjati F, Nikzat N, Mohseni M, et al. Fragile X syndrome screening of families with consanguineous and non-consanguineous parents in the Iranian population. *Eur J Med Genet* 2009; 52(4):170-3.
3. Doherty E, O'Connor R, Zhang A, Lim C, Love JM, Ashton F, et al. Developmental delay referrals and the roles of Fragile X testing and molecular karyotyping: a New Zealand perspective. *Mol Med Rep* 2013; 7(5):1710-4.
4. Maenner MJ, Baker MW, Broman KW, Tian J, Barnes JK, Atkins A, et al. FMR1 CGG expansions: prevalence and sex ratios. *Am J Med Genet B Neuropsychiatr Genet* 2013; 162(5):466-73.
5. Fatima T, Zaidi SA, Sarfraz N, Perween S, Khurshid F, Imtiaz F. Frequency of FMR1 gene mutation and CGG repeat polymorphism in intellectually disabled children in Pakistan. *Am J Med Genet A* 2014; 164(5):1151-61.

6. Chaudhary AG, Hussein IR, Abuzenadah A, Gari M, Bassiouni R, Sogaty S, et al. Molecular diagnosis of fragile X syndrome using methylation sensitive techniques in a cohort of patients with intellectual disability. *Pediatr Neurol* 2014; 50:368-76.
7. Chen X, Wang J, Xie H, Zhou W, Wu Y, Wang J, et al. Fragile X syndrome screening in Chinese children with unknown intellectual developmental disorder. *BMC Pediatr*. 2015; 15:77.
8. Kanwal M, Alyas S, Afzal M, Mansoor A, Abbasi R, Tassone F, et al. Molecular diagnosis of fragile X syndrome in subjects with intellectual disability of unknown origin: implications of its prevalence in regional Pakistan. *PLoS One* 2015; 10:e0122213.
9. Ali EZ, Yakob Y, Md Desa N, Ishak T, Zakaria Z, Ngu LK, et al. Molecular analysis of fragile X syndrome (FXS) among Malaysian patients with developmental disability. *Malays J Pathol* 2017; 39:99-106.
10. Chandrasekara B, Wijesundera S, Chong SS, Perera HN. Prevalence of Fragile X Syndrome among children receiving special education and carrier states in first degree relatives. *Ceylon Med J* 2017; 62(2):92-6.
11. Hadi P, Haghani K, Noori-Zadeh A, Bakhtiyari S. Prevalence of fragile X syndrome among patients with mental retardation in the west of Iran. *Front. Biol*. 2018;13(6):464-8.
12. Salimy Z, Akbari MT, Deilamani FK. Assessment of FMR1 triplet repeats in patients affected with mental retardation, fragile X syndrome and primary ovarian insufficiency. *J Genet*. 2020;99(1):1-5.

## Table S2A: Hagerman Fragile X Checklist scores of 36 Idiopathic ID (Cohort I)

| Participant code | CF01-3 | CF02-3 | CF03-3 | CF04-3 | CF05-3 | CF07-3 | CF08-3 | CF10-3 | CF11-3 | CF 12-3 | CF14-3 | CF15-2 | CF16-2 | CF18-2 | CF19-2 | CF20-3 | CF21-2 | CF22-3 | CF23-2 | CF28-2 | CF31-2 | CF32-2 | CF34-2 | CF35-3 | CF36-2 | CF38-3 | CF39-5 | CF43-3 | CF44-2 | CF45-2 | CF46-2 | CF49-3 | CF50-2 | CF51-3 | CF52-2 | CF54-3 |
|---|---|---|---|---|---|---|---|---|---|---|---|---|---|---|---|---|---|---|---|---|---|---|---|---|---|---|---|---|---|---|---|---|---|---|---|---|
| **Gender** | M | M | M | M | F | M | M | M | M | M | M | F | M | F | M | M | F | M | M | M | M | M | M | M | M | M | M | F | M | F | M | M | F | M | M | M |
| **Age (in years)** | 26 | 22 | 26 | 4 | 8 | 14 | 14 | 4 | 5 | 7 | 16 | 10 | 7 | 4 | 12 | 7 | 10 | 7 | 5 | 14 | 11 | 6 | 10 | 16 | 16 | 9 | 15 | 23 | 11 | 9 | 12 | 7 | 11 | 15 | 10 | 6 |
| **Clinical Features** | **Clinical Score** | | | | | | | | | | | | | | | | | | | | | | | | | | | | | | | | | | | |
| **Intellectual Disability** | 2 | 2 | 2 | 2 | 1 | 2 | 2 | 1 | 2 | 2 | 2 | 1 | 2 | 1 | 1 | 2 | 1 | 2 | 2 | 2 | 1 | 1 | 1 | 2 | 1 | 1 | 2 | 2 | 2 | 2 | 2 | 1 | 1 | 1 | 2 | 2 |
| **Hyperactivity** | 0 | 1 | 0 | 0 | 1 | 1 | 0 | 2 | 2 | 2 | 1 | 0 | 2 | 0 | 2 | 0 | 2 | 2 | 2 | 1 | 2 | 0 | 2 | 2 | 0 | 2 | 0 | 0 | 2 | 1 | 0 | 0 | 2 | 0 | 2 | 0 |
| **Short attention span** | 2 | 2 | 2 | 2 | 2 | 2 | 2 | 2 | 2 | 2 | 1 | 2 | 2 | 2 | 2 | 2 | 1 | 2 | 2 | 2 | 2 | 2 | 2 | 2 | 0 | 2 | 2 | 2 | 2 | 2 | 2 | 0 | 2 | 2 | 2 | 2 |
| **Tactile defensive** | 2 | 2 | 1 | 0 | 2 | 1 | 0 | 2 | 2 | 1 | 1 | 0 | 1 | 0 | 2 | 0 | 0 | 1 | 2 | 0 | 0 | 0 | 1 | 2 | 0 | 0 | 2 | 0 | 2 | 0 | 2 | 0 | 0 | 0 | 0 | 0 |
| **Hand-flapping** | 1 | 0 | 0 | 0 | 0 | 0 | 0 | 0 | 0 | 0 | 0 | 0 | 2 | 0 | 0 | 0 | 2 | 0 | 0 | 0 | 0 | 2 | 0 | 2 | 0 | 0 | 0 | 0 | 2 | 0 | 2 | 0 | 0 | 0 | 0 | 0 |
| **Hand-biting** | 1 | 2 | 1 | 0 | 0 | 0 | 0 | 0 | 0 | 0 | 0 | 0 | 2 | 0 | 0 | 0 | 0 | 0 | 0 | 0 | 0 | 2 | 0 | 0 | 0 | 0 | 1 | 0 | 2 | 0 | 0 | 1 | 0 | 0 | 0 | 0 |
| **Poor eye contact** | 2 | 2 | 2 | 2 | 1 | 2 | 2 | 2 | 2 | 2 | 2 | 2 | 2 | 2 | 0 | 2 | 2 | 2 | 2 | 1 | 2 | 2 | 2 | 2 | 2 | 1 | 2 | 0 | 2 | 1 | 2 | 2 | 2 | 2 | 2 | 2 |
| **Perseverative speech** | 0 | 2 | 2 | 2 | 1 | 2 | 2 | 2 | 0 | 2 | 2 | 2 | 0 | 2 | 2 | 1 | 2 | 2 | 2 | 1 | 2 | 0 | 2 | 0 | 2 | 1 | 2 | 0 | 2 | 0 | 0 | 2 | 2 | 2 | 2 | 2 |
| **Hyperextensible MP Joints** | 0 | 2 | 2 | 0 | 0 | 0 | 0 | 0 | 0 | 0 | 0 | 0 | 0 | 0 | 2 | 0 | 0 | 0 | 0 | 0 | 0 | 0 | 0 | 0 | 0 | 0 | 2 | 0 | 2 | 0 | 2 | 0 | 0 | 2 | 0 | 0 |
| **Large or Prominent Ears** | 2 | 2 | 0 | 2 | 0 | 0 | 0 | 0 | 2 | 0 | 0 | 0 | 0 | 0 | 2 | 0 | 0 | 0 | 0 | 0 | 0 | 2 | 0 | 0 | 0 | 0 | 2 | 0 | 2 | 0 | 0 | 0 | 0 | 0 | 0 | 0 |
| **Large testicles (Macroorchidism)** | 1 | 2 | 0 | 0 | NA | 0 | 0 | 0 | 0 | 0 | 0 | NA | 0 | NA | 0 | 0 | NA | 0 | 0 | 0 | 0 | 0 | 0 | 0 | 0 | 0 | 2 | NA | 0 | NA | 0 | 0 | NA | 0 | 0 | 0 |
| **Simian crease or Sydney line** | 0 | 0 | 0 | 0 | 0 | 0 | 0 | 0 | 0 | 0 | 0 | 0 | 0 | 0 | 0 | 0 | 0 | 0 | 0 | 0 | 0 | 0 | 0 | 0 | 0 | 0 | 0 | 0 | 0 | 0 | 0 | 0 | 0 | 0 | 0 | 0 |
| **Family history** | 0 | 2 | 0 | 0 | 2 | 2 | 2 | 0 | 2 | 0 | 2 | 0 | 0 | 2 | 0 | 0 | 0 | 0 | 0 | 0 | 0 | 2 | 0 | 0 | 0 | 2 | 2 | 2 | 0 | 2 | 0 | 0 | 2 | 0 | 0 | 0 |
| **Total Score** | 13 | 21* | 12 | 10 | 10 | 12 | 10 | 11 | 14 | 11 | 11 | 7 | 13 | 9 | 13 | 7 | 10 | 11 | 12 | 7 | 9 | 13 | 10 | 12 | 5 | 9 | 19* | 6 | 20* | 8 | 12 | 6 | 11 | 9 | 10 | 8 |

**NA, Not Applicable; M, Male; F, Female; * Positive for CGG repeat expansion**

## Table S2B: Hagerman Fragile X checklist scores of 12 ASD probands (Cohort II)

| **Participant code** | **CF06-3** | **CF17-3** | **CF24-2** | **CF26-3** | **CF27-2** | **CF30-3** | **CF33-3** | **CF37-2** | **CF40-1** | **CF41-2** | **CF42-3** | **CF48-2** |
|---|---|---|---|---|---|---|---|---|---|---|---|---|
| Gender | M | M | F | M | M | M | M | M | M | M | F | F |
| Age (in years) | 16 | 7 | 8 | 8 | 9 | 5 | 5 | 13 | 15 | 7 | 7 | 5 |
| **Clinical Features** | **Clinical Score** | | | | | | | | | | | |
| Mental Retardation | 1 | 2 | 1 | 1 | 2 | 2 | 1 | 2 | 2 | 2 | 1 | 2 |
| Hyperactivity | 2 | 2 | 2 | 0 | 2 | 0 | 0 | 0 | 2 | 0 | 2 | 2 |
| Short attention span | 2 | 2 | 2 | 1 | 2 | 2 | 2 | 2 | 2 | 2 | 2 | 2 |
| Tactile defensive | 2 | 0 | 2 | 2 | 1 | 0 | 2 | 0 | 1 | 2 | 2 | 2 |
| Hand-flapping | 0 | 0 | 0 | 1 | 0 | 2 | 2 | 1 | 2 | 0 | 0 | 0 |
| Hand-biting | 2 | 0 | 0 | 1 | 0 | 2 | 0 | 1 | 2 | 0 | 2 | 2 |
| Poor eye contact | 2 | 2 | 1 | 1 | 2 | 2 | 2 | 2 | 0 | 2 | 2 | 2 |
| Perseverative speech | 2 | 2 | 2 | 2 | 2 | 2 | 2 | 2 | 2 | 2 | 2 | 2 |
| Hyperextensible MP Joints | 0 | 0 | 0 | 0 | 0 | 0 | 0 | 0 | 0 | 0 | 0 | 0 |
| Large or Prominent Ears | 0 | 0 | 0 | 0 | 0 | 0 | 0 | 0 | 0 | 0 | 1 | 0 |
| Large testicles (Macroorchidism) | 0 | 0 | NA | 0 | 0 | 0 | 0 | 0 | 0 | 0 | NA | NA |
| Simian crease or Sydney line | 0 | 0 | 0 | 0 | 0 | 0 | 0 | 0 | 0 | 0 | 0 | 0 |
| Family history | 0 | 2 | 0 | 0 | 2 | 2 | 0 | 2 | 0 | 0 | 0 | 0 |
| **Total Score** | **13** | **13** | **10** | **9** | **13** | **14** | **11** | **12** | **13** | **10** | **14** | **14** |

**NA = Not Applicable; M = Male; F=Female**

**Table S2C**: **Hagerman Fragile X Checklist scores of affected relatives of Cohorts I & II**

| **Participant code** | **CF1-4** | **CF2-4** | **CF3-4** | **CF6-4** | **CF6-5** | **CF38-4** | **CF39-1** | **CF39-2** | **CF39-3** | **CF45-1** |
|---|---|---|---|---|---|---|---|---|---|---|
| Gender | M | M | M | F | M | F | F | M | M | F |
| Age (in years) | 24 | 13 | 23 | 14 | 11 | 8 | 45 | 53 | 60 | 31 |
| **Clinical Features** | **Clinical Score** | | | | | | | | | |
| Mental Retardation | 2 | 2 | 2 | 2 | 2 | 2 | 2 | 2 | 2 | 2 |
| Hyperactivity | 0 | 1 | 0 | 2 | 2 | 0 | 1 | 0 | 0 | 0 |
| Short attention span | 0 | 2 | 0 | 2 | 2 | 0 | 1 | 2 | 2 | 0 |
| Tactile defensive | 0 | 2 | 0 | 2 | 2 | 0 | 0 | 0 | 0 | 0 |
| Hand-flapping | 0 | 0 | 0 | 0 | 2 | 0 | 0 | 0 | 0 | 0 |
| Hand-biting | 0 | 2 | 0 | 0 | 0 | 0 | 2 | 0 | 0 | 0 |
| Poor eye contact | 2 | 2 | 2 | 2 | 2 | 1 | 1 | 2 | 2 | 0 |
| Perseverative speech | 2 | 2 | 2 | 0 | 0 | 1 | 2 | 2 | 2 | 2 |
| Hyperextensible MP Joints | 1 | 2 | 0 | 0 | 0 | 0 | 0 | 2 | 2 | 0 |
| Large or Prominent Ears | 2 | 2 | 2 | 0 | 0 | 0 | 2 | 2 | 2 | 0 |
| Large testicles (Macroorchidism) | 2 | 2 | 0 | NA | 0 | NA | NA | 2 | 2 | NA |
| Simian crease or Sydney line | 0 | 0 | 0 | 0 | 0 | 0 | 0 | 0 | 0 | 0 |
| Family history | 2 | 2 | 2 | 2 | 2 | 2 | 2 | 2 | 2 | 2 |
| **Total Score** | **13** | **21*** | **10** | **12** | **14** | **6** | **13*** | **16*** | **16** | **6** |

**NA, Not Applicable; M, Male; F, Female; * CGG repeat expansion**

**Table S3: Menstrual, Reproductive and Hormonal profiles of idiopathic primary ovarian insufficiency probands (Cohort –III)**

| Lab. Ref Code No | Age at Interview | Physical examination | History of Chronic illness, Chemotherapy, Radiotherapy, uterus removal | Age at Menarche | Age at Menopause | Age at Marriage | Type of Amenorrhea | Currently undertaking oral contraceptives/Hormonal treatment | Reproductive history | History of Abortion for the Index cases | FSH (m IU/ml) | Family History of POI (Menopause at or >40 yrs) | Maternal age at Menopause (in Yrs) | Family H/o EM (Menopause at or > 47 yrs) | Family History of MR /Autism/LD | Family History of Infertility | Family history of Dizygous twinning | Family history of Pregnancy loss / Trisomies |
|---|---|---|---|---|---|---|---|---|---|---|---|---|---|---|---|---|---|---|
| POI1-1 | 33 | Normal | No | 14 | 21 | 25 | Secondary | + | Infertile | - | 47.92 | 1 | 46 | 1 | 0 | 1 | 0 | 0 |
| POI1-2 | 32 | Normal | No | 16 | 21 | 23 | Secondary | + | Infertile | - | 42.77 | 1 | 46 | 1 | 0 | 1 | 0 | 0 |
| POI2 | 43 | Normal | No | 15 | 21 | 20 | Secondary | + | Fertile | - | 48.3 | 0 | 49 | 0 | 0 | 0 | 0 | 1 |
| POI3 | 29 | Normal | No | 20 | 26 | 26 | Secondary | + | Infertile | - | 48.83 | 0 | 47 | 1 | 0 | 1 | 0 | 0 |
| POI4 | 38 | Normal | No | 15 | 37 | 20 | Secondary | + | Infertile | - | 87.4 | 0 | NA | 0 | 0 | 0 | 0 | 0 |
| POI5 | 23 | Normal | No | 15 | 22 | 15 | Secondary | + | Infertile | - | >110 | 0 | NA | 0 | 0 | 0 | 0 | 0 |
| POI7 | 25 | Normal | No | 16 | 20 | 17 | Secondary | + | Fertile | + | 53.91 | 0 | 47* | 0 | 1 | 0 | 1 | 0 |
| POI8 | 42 | Normal | No | 16 | 28 | 17 | Secondary | + | Fertile | - | 69.1 | 0 | 49 | 0 | 0 | 1 | 0 | 0 |
| POI9 | 25 | Normal | No | 16 | 23 | 22 | Secondary | + | Infertile | - | 45.98 | 0 | 42 | 0 | 0 | 0 | 0 | 0 |
| POI10 | 34 | Normal | No | 16 | 33 | NM | Secondary | + | Not married | - | 72.4 | 0 | 50 | 0 | 0 | 1 | 0 | 0 |
| POI11-1 | 21 | Normal | No | 16 | 18 | 20 | Secondary | + | Infertile | - | 51.66 | 1 | 40 | 0 | 0 | 1 | 0 | 0 |
| POI11-2 | 26 | Normal | No | 16 | 18 | 17 | Secondary | + | Infertile | - | 28.62 | 1 | 40 | 0 | 0 | 1 | 0 | 0 |
| POI12 | 26 | Normal | No | 19 | 23 | 20 | Secondary | + | Infertile | - | 62.2 | 1 | 48 | 0 | 0 | 0 | 0 | 0 |
| POI13-1 | 21 | Normal | No | 16 | 19 | 21 | Secondary | + | Infertile | - | 76.38 | 1 | 46 | 1 | 0 | 1 | 0 | 0 |
| POI13-2 | 27 | Normal | No | 16 | 19 | 17 | Secondary | + | Infertile | - | 76 | 1 | 46 | 1 | 0 | 1 | 0 | 0 |
| POI 15-1 | 42 | Normal | No | 16 | 38 | 22 | Secondary | - | Fertile | - | 62 | 1 | NA | 1 | 0 | 0 | 0 | 0 |
| POI 15-2 | 19 | Normal | No | 16 | 18 | NM | Secondary | + | Not married | - | 71 | 1 | 22 | 1 | 0 | 0 | 0 | 0 |

*Menopause due to surgery

**Table S4: Frequency distribution of chromosomes by CGG repeats among relatives of idiopathic ID**

| CGG repeat Number | Affected relatives | | | | Unaffected relatives | | | |
|---|---|---|---|---|---|---|---|---|
| | Males (5) | | Females (3) | | Males (21) | | Females (37) | |
| | **n** | **%** | **n** | **%** | **n** | **%** | **n** | **%** |
| 20 | | | 1 | 16.67 | | | | |
| 21 | | | | | | | 1 | 1.35 |
| 22 | | | | | | | 1 | 1.35 |
| 23 | | | | | 1 | 4.76 | 2 | 2.70 |
| 24 | | | | | | | 3 | 4.05 |
| 25 | | | | | | | 2 | 2.70 |
| 27 | | | 4 | 66.67 | 1 | 4.76 | | |
| 29 | 1 | 20 | | | 3 | 14.29 | 25 | 33.78 |
| 30 | | | | | 9 | 42.86 | 28 | 37.84 |
| 31 | | | | | | | 2 | 2.70 |
| 32 | 2 | 40 | | | 1 | 4.76 | 1 | 1.35 |
| 33 | | | | | 1 | 4.76 | | |
| 34 | | | | | | | 1 | 1.35 |
| 36 | | | | | 1 | 4.76 | 2 | 2.70 |
| 38 | | | | | | | 1 | 1.35 |
| 39 | | | | | 3 | 14.29 | 1 | 1.35 |
| 44 | | | | | 1 | 4.76 | 1 | 1.35 |
| 62 | | | | | | | 1 | 1.35 |
| 82 | | | 1 | 16.67 | | | | |
| 94 | | | | | | | 1 | 1.35 |
| PFM | | | | | | | 1 | 1.35 |
| ≥ 200 | 2 | 40 | | | | | | |
| **Total** | **5** | **100** | **6** | **100** | **21** | **100** | **74** | **100** |

**Table S5**: **Distribution of chromosomes by number of CGG repeats among ASD probands and their relatives**

| CGG Repeat Number | ASD Probands | | | | Affected relatives | | | | Unaffected relatives | | | |
|---|---|---|---|---|---|---|---|---|---|---|---|---|
| | Males (9) | | Females(3) | | Males (1) | | Females (1) | | Males (5) | | Females (11) | |
| | n | % | n | % | n | % | n | % | n | % | n | % |
| 23 | 0 | 0 | 0 | 0 | 0 | 0 | 0 | 0 | 1 | 20 | 0 | 0 |
| 28 | 0 | 0 | 3 | 50 | 0 | 0 | 0 | 0 | 0 | 0 | 0 | 0 |
| 29 | 6 | 66.67 | 0 | 0 | 1 | 100 | 2 | 100 | 2 | 40 | 10 | 45.45 |
| 30 | 3 | 33.33 | 3 | 50 | 0 | 0 | 0 | 0 | 2 | 40 | 7 | 31.82 |
| 31 | 0 | 0 | 0 | 0 | 0 | 0 | 0 | 0 | 0 | 0 | 2 | 9.09 |
| 32 | 0 | 0 | 0 | 0 | 0 | 0 | 0 | 0 | 0 | 0 | 2 | 9.09 |
| 42 | 0 | 0 | 0 | 0 | 0 | 0 | 0 | 0 | 0 | 0 | 1 | 4.55 |
| **Total** | 9 | 100 | 6 | 100 | 1 | 100 | 2 | 100 | 5 | 100 | 22 | 100 |

**Table S6: Distribution of chromosomes by number of CGG repeats among idiopathic POI probands and their affected relatives**

| CGG repeat Number | Number of Chromosomes | Percentage (%) |
|---|---|---|
| 25 | 2 | 5.88 |
| 29 | 12 | 35.29 |
| 30 | 13 | 38.24 |
| 31 | 1 | 2.94 |
| 32 | 2 | 5.88 |
| 35 | 1 | 2.94 |
| 36 | 2 | 5.88 |
| 37 | 1 | 2.94 |
| **Total** | **34** | **100** |

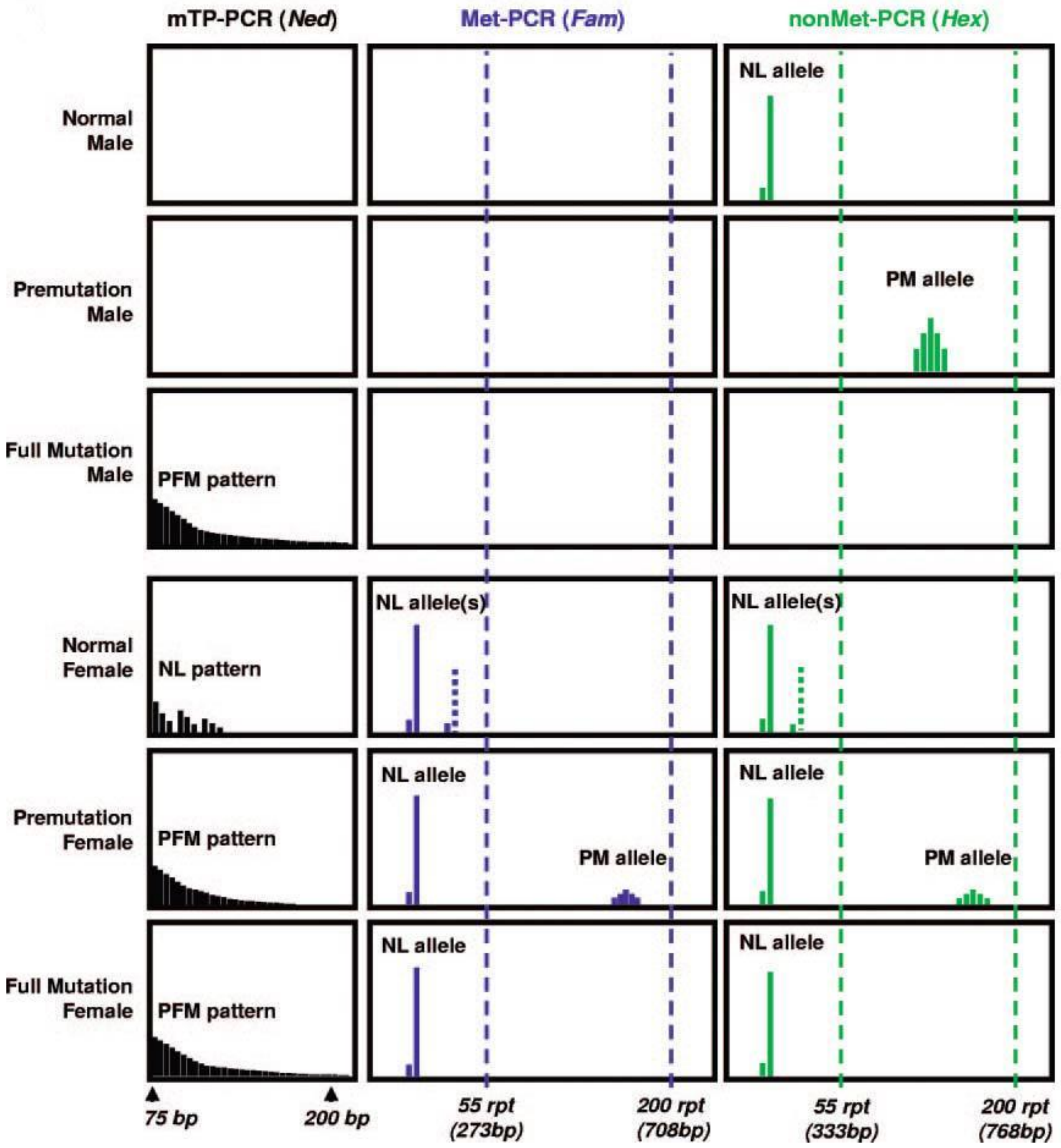


**Figure S1: Schematic representation of normal (NL), premutation (PM), full mutation (FM), and pre/full mutation (PFM) ms-PCR products by GeneScan fragment analysis (adapted from Zhou et al., 2006).**
