# Supplementary material for: FMR1 gene CGG repeat distribution among the three individual cohorts with intellectual disability, autism, and primary ovarian insufficiency from Tamil Nadu, Southern India
Source: Adv Genet (Hoboken). 2021 May 28;2(2):e10048. doi: 10.1002/ggn2.10048 (PMC9744524; doi:10.1002/ggn2.10048)
Supplement: Supplementary file 2 — Supplementary TPR File [file GGN2-2-e10048-s002.pdf]

ADVANCED GENETICS
Open Access

FMR1 gene CGG repeat distribution among the three individual cohorts with intellectual disability, autism and primary ovarian insufficiency from Tamil Nadu, Southern India

Nagarathinam Indhumathi, Samuel S Chong, B K Thelma, Justin Margret Jeffrey, Venkataraman Vishwanathan, Natarajan Padmavathy Karthikeyen, C R Srikumari Srisailapathy*

*Corresponding

Review timeline:		
	Date Submitted:	29-Sep-2020
	Editorial Decision:	31-Dec-2020 Major Revision
	Revision Received:	17-Feb-2021
	Accepted in principle:	28-Apr-2021
	Accepted:	10-May-2021

Editor: Myles Axton

Initial Editorial Evaluation	30-Sep-2020

Summary

The spectrum of CGG repeats in FMR1 was examined by PCR screening in South Indian cohorts ascertained by diagnoses of intellectual disability (ID), autism spectrum (ASD) or premature ovarian failure (POF). 36 ID (10% males had expansions), 12 ASD, 13 POF screened for FMR1 CGG repeats. Frequency is high but sample is small and within the range internationally, and comparable to reference [26] (South India 1998). Hagerman FXC syndromic score was useful in screening before test and was also applied to affected and unaffected relatives of the FRX ID cases and the autism cohort (Appendix 1A-C). Interesting implications might be the risk to carriers with 55-200 CGG for neurodegenerative adult onset disorder FXTAS. What are the implications of South Indian population structure and founder effects for population frequency distribution of FMR1 repeat number?

Scope

Do the research, methods or topics fit within the aims of this, or another journal?

This is a regional study in line with international and regional findings, with exceptionally well documented clinical genetics. It is certainly useful in building clinical testing capacity in India, and given the recent interest in the clinical utility of using founder effects and population structure in India to guide testing for FMR1 repeats in a founder population with apparent high repeat carrier frequency.

What is already known in this area and related fields?

Ref [1] Normal 5-45 CGG repeats are generationally stable, 45–55 gray zone associated with expansion, 55–200 premutation associated with a range of symptoms: FXTAS and POF, >200 CGG associated with hypermethylation and FRX.

Ref. [26] FRX diagnosis in 7% of 98 unclassified mental retardation in South India. POF FMR1 CGG repeat carrier frequency [the authors 18]. Ref [21] 20% of POF are FMR1 premutation carriers.

1st Peer Review	09-Oct-2020

Reviewer #1

1.0 The authors have reported on their screening studies in 3 populations in India including those with ID, those with POF and those with idiopathic ASD. Only the ID group has a significant number of individuals with FXS, specifically 10.34% which is high compared to studies outside of India but consistent with other reports of India that was nicely put in a table. No positive cases were found in the ASD group of only 12 patients and in the POF group of only 13 females and this is not surprising since their numbers are so small. This paper has valuable data that add to the field because few if any studies have been done in Chennai or Southern India. So, there are a few issues that need to be clarified before publication.

1.1 It is important that their terms are correct. For instance, the write out of FXTAS is fragile X-associated tremor ataxia syndrome so they need to insert the hyphen after associated. The term FXPOF has been replaced by FXPOI and this is spelled out as fragile X-associated primary ovarian insufficiency (not failure).

1.2 The abstract and the introduction have to be consistent in saying that fragile X syndrome is the most common genetic cause of ID because one says this but the other says it is the second most common cause of ID. Also, all of the findings and patient numbers need to be expressed in the abstract.

1.3 The term PFM needs to be spelled out when first used. I think they mean PM and FM as they say in other cases but why this is abbreviated differently, I am not sure. In the pedigree CF44-1 is noted to be just a carrier so maybe she just has the premutation, but this needs to be explained. They also need to write out HFXC which I think means Hagerman Fragile X checklist, the first time it is used.

1.4 They can eliminate tables 3,4 and 5 in appendix because there are too many tables, and these are not meaningful for those that are fragile X negative because when the proband is negative it is not worthwhile to do cascade testing through the family.

1.5 the sentence "Among women with POF it is estimated that 20% are FX premutation carriers" because this sentence contradicts the 2 sentences before it. I think the authors meant to say that Approximately 20% of premutation carriers develop FXPOI because this is what the reference that they cited says (ref 21).

1.6 The title for table 1 should clarify that the prevalence figures are for FXS in those with ID in the Indian population.

1.7 The premutation is defined here as 56 to 200 but it should be 55 to 200 CGG repeats. Also, they need to spell premutation as one word throughout the paper and also in the figures.

1.8 Some corrections in the English are needed such as "sustained attentions" should be sustained attention.

Overall this is an interesting study showing the high prevalence of fragile X mutations in Southern India and the importance of cascade testing.

1st Editorial Decision	31-Dec-2020

Editorial decision: Major Revision and re-review

Editor's understanding of the review

Reviewer #1 recommends Major Revision

Reviewer comments	Editor recommendation
1.7 The premutation is defined here as 56 to 200 but it should be 55 to 200 CGG repeats.	ED1 Please correct this definition and if there are any repeat calls of 55 or 56, reclassify these cases throughout the article.
1.4 They can eliminate tables 3,4 and 5 in appendix because there are too many tables, and these are not meaningful for those that are fragile X negative because when the proband is negative it is not worthwhile to do cascade testing through the family.	ED2 The journal allows the authors any number of supplementary tables. However, Reviewer #1 is right that it is not worth tabulating cascade testing of fragile X negative probands. The number tested can be mentioned in the text.
1.0 This paper has valuable data that add to the field because few if any studies have been done in Chennai or Southern India.	ED3 It would therefore be worth expanding the discussion or tabulation of repeat distributions in FXS ID or developmental delay disability cases by Indian regions and in South Asian populations in other countries. How typical is the population of Chennai, Tamil Nadu, South India?
1.1 It is important that their terms are correct.	ED4 Please ensure that all terms and their abbreviations are consistent with HPO and current genetic diagnostic criteria (for example ACMG).

Author’s Response to 1st Review	17-Feb-2021

Reviewer #1

1.0 The authors have reported on their screening studies in 3 populations in India including those with ID, those with POF and those with idiopathic ASD. Only the ID group has a significant number of individuals with FXS, specifically 10.34% which is high compared to studies outside of India but consistent with other reports of India that was nicely put in a table. No positive cases were found in the ASD group of only 12 patients and in the POF group of only 13 females and this is not surprising since their numbers are so small. This paper has valuable data that add to the field because few if any studies have been done in Chennai or Southern India. So, there are a few issues that need to be clarified before publication.

We thank the reviewer for the valuable comments. The clarifications for the comments are given below and we have also pasted the parts that are edited in the running text for easy verification.

1.1 It is important that their terms are correct. For instance, the write out of FXTAS is fragile X-associated tremor ataxia syndrome so they need to insert the hyphen after associated. The term FXPOF has been replaced by FXPOI and this is spelled out as fragile X-associated primary ovarian insufficiency (not failure).

Ans: The hyphen has been inserted and the term FXPOF is now replaced with FXPOI throughout the text and tables.

“Individuals with a PM allele will be asymptomatic but may develop late onset conditions such as fragile X associated - tremor ataxia (FXTAS) in both males and females, or fragile X associated - premature ovarian insufficiency (FXPOI) in females.”

1.2 The abstract and the introduction have to be consistent in saying that fragile X syndrome is the most common genetic cause of ID because one says this but the other says it is the second most common cause of ID. Also, all of the findings and patient numbers need to be expressed in the abstract.

Ans: We have now consistently mentioned that ‘fragile X syndrome is the most common genetic cause of ID’ and removed the phrase ‘the second most common cause of ID’ is also removed. All the finding of the study and the patient numbers now reflect in the abstract. The revised abstract now reads as follows:

“Three (CF2-3, 39-5, 44-2) out of 29 males had full mutation alleles accounting for 10.34% of FXS among idiopathic ID. One of them was a mosaic for CGG repeats with both premutation and full mutation alleles.”

We also added an additional sentence in the abstract as follows:

“A cascade testing that begins with checklist evaluation prior to DNA analysis will be cost- effective for establishing early diagnosis in south India. With the huge disease burden, there is a need for the establishment of the molecular diagnostics and self-help groups for fragile X syndrome.”

1.3 The term PFM needs to be spelled out when first used. I think they mean PM and FM as they say in other cases but why this is abbreviated differently, I am not sure. In the pedigree CF44-1 is noted to be just a carrier so maybe she just has the premutation, but this needs to be explained. They also need to write out HFXC which I think means Hagerman Fragile X checklist, the first time it is used.

Ans: The term PFM notates the mosaic status due to the occurrence of both premutation and full mutations in the same individual. This is the reason we have used the acronym PFM. HFXC is also expanded when it occurs for the first time in the manuscript. The edited portion is pasted below:

“Proband’s mother (CF 44-1; II-2) had both normal allele and a mosaic of premutation and full mutation alleles that is notated as PFM (pre/full mutation) genotype [29/164 or >200] (Fig. 3B; Panel-2). However, she did not have any clinical manifestation.”

“Based on the Wechsler Adult Performance Intelligence Scale his IQ was 45 (moderate ID) and Hagerman Fragile X score checklist (HFXC) score was 19.”

1.4 They can eliminate tables 3,4 and 5 in appendix because there are too many tables, and these are not meaningful for those that are fragile X negative because when the proband is negative it is not worthwhile to do cascade testing through the family.

Ans: We have now eliminated the appendix tables 3, 4 and 5. However we have added a table that presents the prevalence of fragile X syndrome among ID in world populations. This may be included if the reviewers opine this additional information is useful.

Table 1: Studies reporting prevalence of fragile X syndrome in the world population.

S. No Ethnicity Sample Size Methodology Frequency Reference

1. China 50 PCR 2% [39]

2. Iran 508 PCR; Southern Blotting 6.3% [40]

3. New Zealand 2046 Fluorescent PCR; Southern blotting 0.6% [41]

4. Marshfield 8469 PCR 0.3% [42]

5. Pakistan 333 MS PCR; Southern Blotting 4.8% [43]

6. Saudi Arabia 63 ID (53 M & 10 F) MS PCR; Southern blotting 17% [44]

7. China 553 TP-PCR 0.93% [45]

8. Pakistan 395 Conventional PCR; Southern Blotting 3.3% [46]

9. Malaysia 1245 MS PCR 3.5% [47]

10. Sri Lanka 850 TP PCR; MS PCR; Southern Blot 1.3% [48]

11. West Iran 200 Cytogenetic and DNA sequencing 8% [49]

12. Iran 656 (449 M & 207 F) PCR; TP PCR; MS MLPA 10.36% [12]

M – Males; F – Females

1.5 the sentence "Among women with POF it is estimated that 20% are FX premutation carriers" because this sentence contradicts the 2 sentences before it. I think the authors meant to say that Approximately 20% of premutation carriers develop FXPOI because this is what the reference that they cited says (ref 21).

Ans: Thank you for this comment we have now amended this sentence as "Approximately 20% of premutation carriers develop FXPOI". It reads in the manuscript as follows:

"A review of several screening studies of POI cohorts shows that FX premutation occurrence in familial cases is high (9.5%) compared to sporadic cases, where the reported frequency is 3% [19-20]. Approximately 20% of premutation carriers develop FXPOI [21]."

1.6 The title for table 1 should clarify that the prevalence figures are for FXS in those with ID in the Indian population.

Ans: Yes, we agree. The modified title reads as follows:

"Table 1: Studies reporting prevalence of fragile X syndrome in those with ID in the Indian population"

1.7 The premutation is defined here as 56 to 200 but it should be 55 to 200 CGG repeats. Also, they need to spell premutation as one word throughout the paper and also in the figures.

Ans: Yes, this has been now changed as follows in the revised manuscript.

"The CGG triplet is usually composed of 5–44 repeats (normal) or 45-54 repeats (intermediate or gray zone - GZ), allowing transcription and translation of the FMR1 gene. Normal alleles are usually transmitted stably over generations. Intermediate alleles may or may not reveal intergenerational instability and are considered a possible risk factor for the CGG repeat expansion [2]. When the CGG repeat expands between 55 and 200 (premutation - PM), the gene continues to transcribe (more) mRNA even though the premutated repeats become meiotically unstable [3]."

1.8 Some corrections in the English are needed such as "sustained attentions" should be sustained attention.

Ans: This has now been taken care and reads as follows:

"FXS is also associated with risk for deficits in other domains, including executive function, sustained attention, working memory, and social function even in those without mental retardation [4]."

Overall this is an interesting study showing the high prevalence of fragile X mutations in Southern India and the importance of cascade testing.

Other minor grammatical changes are marked in red in the running text itself.

2nd Peer Review	22-Feb-2021 to 27-Apr-2021

Reviewer 1 recommends Accept

the Authors have carried out a detailed revision of this paper and it is now ready for publication.

Reviewer 2 recommends Minor Revision

The manuscript is well written in terms of fluency and scientific content. The authors address their small sample size and reason their findings appropriately. However, there are few changes that are suggested:

2.1 The second family in the ID cohort CF39, sister of proband with 30/62 repeats as well as the maternal uncle with 30 repeats have ID indicating there is possibility of another genetic etiology, though the authors mention the maternal uncle should be investigated further, such study is also warranted in the sister.

2.2 The pedigree of same family, CF39 should show the III.2 individual (sister) to be affected

2.3 No individuals from the premature ovarian failure show expanded repeat size, in the earlier publication by the same group, 1 in 353 prevalence was reported for POI with expanded repeats. This should be further discussed with consideration to the statement from the manuscript Introduction "the women with a family history of POI or elevated levels of follicle-stimulating hormone (FSH) before the age of 40 years without a known cause should be tested to rule out PM positivity as a cause of ovarian insufficiency".

2.4 There is no conclusion section in the manuscript, if that is the journal style, please include a concluding paragraph at the end of the discussion.

Reviewer 3 recommends Accept

The authors have adequately addressed prior comments from the reviewers. This study is a useful source of data to the field. The discussion on relevance of the study to the Indian population is well presented.

2nd Editorial Decision	28-Apr-2021

The manuscript has now been seen by the original reviewer and two additional reviewers, and based on these new reviews, we have now decided to accept the revised manuscript in principle, subject to incorporating the improvements recommended by Reviewer 2 and the attached formatting requirements.

Author's Response to 2nd Review	8-May-2021

**Reviewer comments**	**Editor recommendation**	**Author reply**	**Changes to Manuscript**
2.1 The second family in the ID cohort CF39, sister of proband with 30/62 repeats as well as the maternal uncle with 30 repeats have ID indicating there is possibility of another genetic etiology, though the authors mention the	ED5 Sister CF39-5 III-1 (of proband CF39-5 III-2) has PM but no disability, Mother CF-39-1 II-8 has PM and disability, and uncle CF39-3 II-6 has 30 CGG and ID.<br><br>Comment on whether you think CF39-1 and CF39-3	We agree to the reviewer's comment and editor's recommendation.<br><br>CF39-1 and CF39-3 might be sharing a non CGG expansion genetic cause for their shared phenotypes. However, more detailed	The proband's two maternal uncles (CF39-2, CF39-3) have ID with similar facial characteristics and macroorchidism. However, one of them (CF39-2; II-7, 53 years) carries a FM allele of >200 repeats with severe ID (**Fig. 2B; Panel-3**), while the

maternal uncle should be investigated further, such study is also warranted in the sister.	share a non CGG expansion genetic cause of their shared phenotypes or what other future investigation of this pedigree (not just CF39-3) might be warranted.<br><br>For example, are there point mutations in *FMR1* that have been published that might be hemizygous in CF39-1 in compound heterozygosity in CF39-1?<br><br>Thirdly might there be a non *FMR1* mutation also segregating in this pedigree?	future clinical investigation for other psychiatric disorders might be warranted to resolve the phenotypes.<br>Similarly, CF39-5 should be followed up in future for any FXTAS and FXPOI manifestations with age.	other uncle (CF39-3; II-6, 60 years) has only a normal allele of 30 CGG repeats (**Fig. 2B; Panel-4**); his intellectual disability despite not having a FM allele merits further investigation for other psychiatric disorders to achieve finer resolution of the phenotype. CF39-1 and CF39-3 might be sharing a non CGG expansion genetic cause for their shared phenotypes. Similarly, the proband's PM carrier sibling CF39-5 should be followed up in future for any FXTAS and FXPOI manifestations with age.
2.2 The pedigree of same family, CF39 should show the III.2 individual (sister) to be affected	ED6 III-2 is the male proband, so Reviewer 2 may have been confused by the figure legends to Fig 2B and Fig 2A as well as the text account of CF-39 pedigree. Check text and both legends carefully so that they are simple to understand and consistent.<br><br>In the text the authors state "His asymptomatic sister (CF39-4; III-1) is a PM carrier with 30/62 repeats (Fig. 2B; Panel-5)."<br><br>In Fig 2A the pedigree of the male proband CF39-5 (CF-39 III-2). His sister (CF39-4; III-1) is consistently shown as a PM carrier with 30/62.<br><br>Fig 2B gene scan legend reads "proband's mother CF39-1 (Panel 2) his elder sister CF39-4 (Panel 5) have both normal and PM allele".	Yes, III-2 is the male proband belonging to the CF39 family.<br><br>To make it more simple to understand as suggested by the editor we have reframed the sentences in the legend using terms such as 'affected' and 'asymptomatic'.<br><br>The crosshatching has been given for the mother who is affected.<br><br>The same correction has also been adapted in the text.	**Figure 2B:** GeneScan results from fluorescent ms-PCR assay for CF39 family. The proband CF39-5 (Panel 1) and his affected younger maternal uncle CF39-2 (Panel 3) were found to have FM allele (>200); proband's affected mother CF39-1 (Panel 2) his asymptomatic elder sister CF39-4 (Panel 5) have both normal and PM allele; proband's affected elder maternal uncle CF39-3 (Panel 4) has a normal allele.
2.3 No individuals from the premature ovarian failure show expanded repeat size, in the earlier publication by the same group, 1 in 353 prevalence was reported for POI with expanded repeats. This should be further discussed with	ED7 Explain that finding no intermediate, PM and FM alleles in 8 familial and 9 sporadic cases was consistent with the frequencies expected from the cited references in the introduction:	The carrier frequency [1 in 353] in our own study [Reference 22] was with respect to 705 normal women and not women with POI as remarked by the Reviewer 2. Hence now we have removed the reference 22 in between the POI	Neither the 13 POI probands nor their four affected relatives showed any CGG expansion in our study. Since the sample size is small, we are not able to come up with a confident statistical correlation. A review of several screening

consideration to the statement from the manuscript Introduction "the women with a family history of POI or elevated levels of follicle-stimulating hormone (FSH) before the age of 40 years without a known cause should be tested to rule out PM positivity as a cause of ovarian insufficiency".	Expect 2 PM in 17 POI women [10]<br>OR expect 0.76 PM in 8 familial<br>and 0.27 PM in 9 sporadic [23-24]	studies but we have now pasted it at the end of the paragraph [to remove confusion]. However, to emphasize the need for screening women with POI we have cited our study on normal women that was conducted from the same region where we have ascertained the POI.	studies of POI cohorts shows that FX premutation occurrence in familial cases to be high (9.5%) compared to sporadic cases, where the reported frequency is 3% [22-23]. Approximately 20% of premutation carriers develop FXPOI [24]. Timely information about the individual's carrier status will help them make informed reproductive decisions in the light of the genetic risk [11]. However, in our earlier study from the same region, screening of 705 normal women for premutation showed a carrier frequency of 1 in 353 (0.14%) [25].
2.4 There is no conclusion section in the manuscript, if that is the journal style, please include a concluding paragraph at the end of the discussion.	ED8 A Conclusion or concluding paragraph in the Discussion section is at the authors' discretion.	We opt to leave it as it is, since a kind of conclusion is embedded in the last paragraph.	

Reviewer 2 recommends Minor Revision

The manuscript is well written in terms of fluency and scientific content. The authors address their small sample size and reason their findings appropriately. However, there are few changes that are suggested:

2.1 The second family in the ID cohort CF39, sister of proband with 30/62 repeats as well as the maternal uncle with 30 repeats have ID indicating there is possibility of another genetic etiology, though the authors mention the maternal uncle should be investigated further, such study is also warranted in the sister.

Reply: We agree to the reviewer's comment and editor's recommendation.

CF39-1 and CF39-3 might be sharing a non CGG expansion genetic cause for their shared phenotypes. However, more detailed future clinical investigation for other psychiatric disorders might be warranted to resolve the phenotypes. Similarly, CF39-5 should be followed up in future for any FXTAS and FXPOI manifestations with age.

The text in the fourth paragraph of the results section now reads as follows:

The proband's two maternal uncles (CF39-2, CF39-3) have ID with similar facial characteristics and macroorchidism. However, one of them (CF39-2; II-7, 53 years) carries a FM allele of >200 repeats with severe ID (**Fig. 2B; Panel-3**), while the other uncle (CF39-3; II-6, 60 years) has only a normal allele of 30 CGG repeats (**Fig. 2B; Panel-4**); his intellectual disability despite not having a FM allele merits further investigation for other psychiatric disorders to achieve finer resolution of the phenotype. CF39-1 and CF39-3 might be sharing a non CGG expansion genetic cause for their shared phenotypes. Similarly, the proband's PM carrier sibling CF39-5 should be followed up in future for any FXTAS and FXPOI manifestations with age.

2.2 The pedigree of same family, CF39 should show the III.2 individual (sister) to be affected.

Reply: III-2 is the male proband belonging to the CF39 family. To make it more simple to understand as suggested by the editor we have reframed the sentences in the legend using terms such as 'affected' and 'asymptomatic'.

The crosshatching has been given for the mother who is affected. The same correction has also been adapted in the text.

The legend for figure 2B is now changed as:

Figure 2B: GeneScan results from fluorescent ms-PCR assay for CF39 family.
The proband CF39-5 (Panel 1) and his affected younger maternal uncle CF39-2 (Panel 3) were found to have FM allele (>200); proband's affected mother CF39-1 (Panel 2) his asymptomatic elder sister CF39-4 (Panel 5) have both normal and PM allele; proband's affected elder maternal uncle CF39-3 (Panel 4) has a normal allele.

2.3 No individuals from the premature ovarian failure show expanded repeat size, in the earlier publication by the same group, 1 in 353 prevalence was reported for POI with expanded repeats. This should be further discussed with consideration to the statement from the manuscript Introduction "the women with a family history of POI or elevated levels of follicle-stimulating hormone (FSH) before the age of 40 years without a known cause should be tested to rule out PM positivity as a cause of ovarian insufficiency".

Reply: The carrier frequency [1 in 353] in our own study [Reference 22] was with respect to **705 normal women** and not women with POI as remarked by the Reviewer 2. Hence now we have removed the reference 22 in between the POI studies but we have now pasted it at the end of the paragraph [to remove confusion]. However, to emphasize the need for screening women with POI we have cited our study on normal women that was conducted from the same region where we have ascertained the POI.

The modified text in the fifth paragraph of the discussion section now reads as:

Neither the 13 POI probands nor their four affected relatives showed any CGG expansion in our study. Since the sample size is small, we are not able to come up with a confident statistical correlation. A review of several screening studies of POI cohorts shows that FX premutation occurrence in familial cases to be high (9.5%) compared to sporadic cases, where the reported frequency is 3% [22-23]. Approximately 20% of premutation carriers develop FXPOI [24]. Timely information about the individual's carrier status will help them make informed reproductive decisions in the light of the genetic risk [11]. However, in our earlier study from the same region, screening of 705 normal women for premutation showed a carrier frequency of 1 in 353 (0.14%) [25].

2.4 There is no conclusion section in the manuscript, if that is the journal style, please include a concluding paragraph at the end of the discussion.

Reply: We opt to leave it as it is, since a kind of conclusion is embedded in the last paragraph.

Final Editorial Decision	10-May-2021

The authors have addressed and incorporated the second reviewer's points and the manuscript has been accepted for publication.
